# Appendicular mass imitating a malignant cecal tumor on f18-FDG PET/CT study: a case report

**DOI:** 10.4076/1757-1626-2-8420

**Published:** 2009-08-04

**Authors:** Ömer Günal, Semih Doğan, Emin Gürleyik

**Affiliations:** 1 Department of General Surgery, Düzce University, School of MedicineKonuralp, Düzce, 81620Turkey; 2 Department of Nuclear Medicine, Düzce University, School of MedicineKonuralp, Düzce, 81620Turkey

## Abstract

Fluoro-Deoxy-Glucose Positron Emission Tomography/Computerized Tomography scan is a very useful method in the diagnosis and follow-up of gastrointestinal malignancies, although it may cause confusion in differential diagnosis.

We present a 48-year-old man admitted with a right lower quadrant mass. Upon an unyielding colonoscopy due to inability to pass beyond the hepatic flexure, a Fluoro-Deoxy-Glucose Positron Emission Tomography/Computerized Tomography strongly suggested a right colonic or cecal malignancy. Eventual laparatomy revealed a periappendiceal plastron due to appendicitis that was subsequently proven histological diagnosis.

Although Fluoro-Deoxy-Glucose Positron Emission Tomography/Computerized Tomography is a reliable diagnostic tool for colonic malignancies, it can misdiagnose such masses due to inflammatory process around the cecum.

## Introduction

Positron emission tomography (PET), using 18F-fluoro-2-deoxy-d-glucose (FDG) as a tracer, is preferentially taken up by most malignant neoplasms with altered glucose metabolism. In recent years, the fusion of images from FDG-PET with those of computed tomography (PET/CT) has aided in the precise localization of increased FDG uptake sites, which may occur not only in malignant tumors but also in areas of inflammation and in areas of physiologic glucose uptake [1]. Lim [2] reported two false positive PET/CT cases due to chronic inflammation and suture granuloma.

We aimed to discuss the reliability and applicability of PET/CT scan when diagnosing colonic malignancies in the event of intra-abdominal inflammatory processes such as appendicular plastrons in context with a case.

## Case presentation

A 48-year-old Caucasian Turkish male was admitted to ER with right lower quadrant pain, and accompanying fever, sweating, constipation and significant weight loss (>10%) during the last two months. Physical examination revealed normal findings apart from painful right lower quadrant palpable mass. Routine biochemical analysis and CBC were unremarkable. CT examination revealed a 3 × 2 × 2 cm size conglomerated solid mass with no contrast localization. The patient improved and passed flatus after five days of supportive therapy with IV fluid replacement and antibiotic therapy and discharged from hospital. It was considered as a plastroned appendicitis (peri-appendiceal mass) and periodic ultrasonographic examinations (USG) were planned for follow-up. However, the USG two weeks after CT scan, revealed a 5 × 3 cm size irregular contoured, heterogenous caecal mass that was neighboring with the urinary bladder. We also proved the increase in size of the mass by physical examination. This finding has led us to the primary diagnosis of a colonic malignancy.

**Figure 1.**
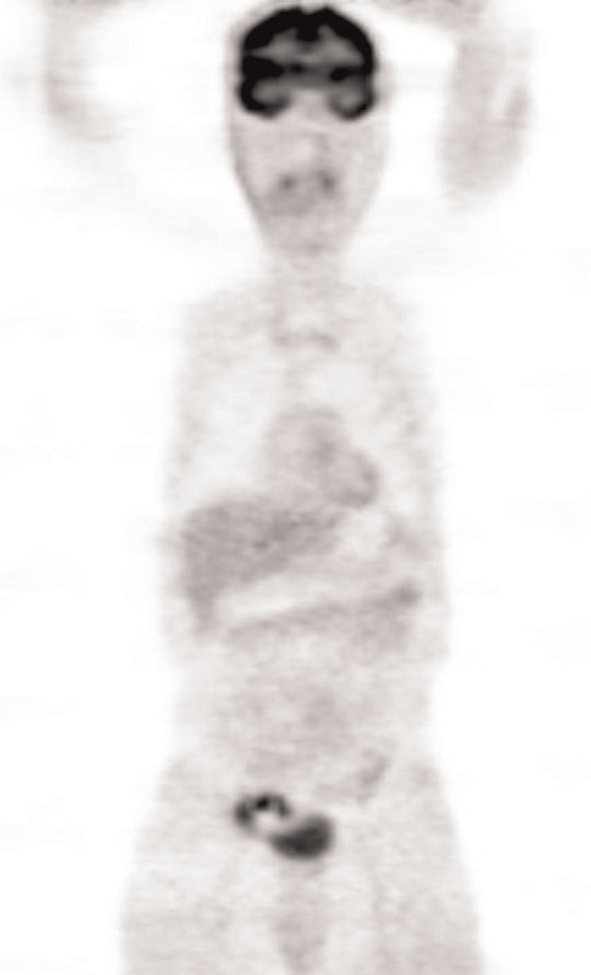
Right lower quadrant mass and urinary bladder activity in coronal section.

**Figure 2.**
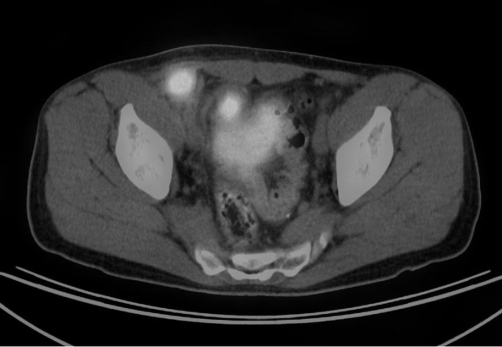
Right lower quadrant mass in PET/CT scan in axial section.

In an attempt to rule out a colonic malignancy, an urgent colonoscopy was performed, which was unyielding due to inability to pass beyond the hepatic flexure. Therefore, we performed an FDG PET/CT study to aid in the differential diagnosis, which showed markedly increased glucose metabolism in two masses localized in the right lower quadrant which were 2 cm in diameter (Figure 1,2).

**Figure 3.**
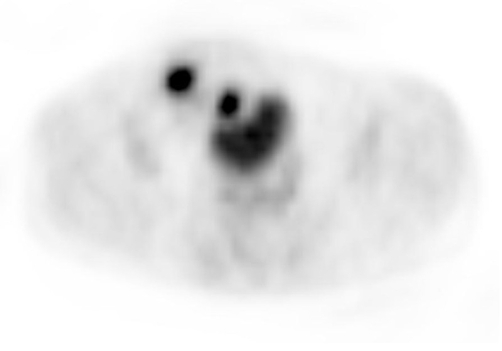
Right lower quadrant mass in axial PET scan.

The high SUV values (early: 10.8, late: 14.7) suggested that these masses were most likely related with malignant tissue (Figure 3). Later in several days, the size of mass has decreased on physical and USG examinations, therefore we decided to follow-up conservatively. In two months, the mass has reduced to nearly an unpalpable size. Surgical exploration revealed a peri-appendicular mass consisting mainly of the omentum at the tip of the appendix. Patient underwent appendectomy and resection of neighboring mass. Histopathological examination of specimen confirmed the diagnosis of appendicitis. He had an uneventful postoperative period and discharged from the hospital at the end of the first week.

## Discussion

Combined PET/CT imaging is a non-invasive nuclear medicine procedure that is gaining increasing application in oncology as a standard modality in the diagnosis of occult cancers, restaging and monitoring of therapeutic efficacy. Metabolic abnormalities detected on PET images can be precisely localized anatomically by hardware fusion with the CT images obtained in the same sitting. Tissues with increased glucose metabolism, such as malignant lesions, appear as areas of increased activity on PET scans due to the trapped FDG within the cells. We observed a strong FDG activity in right lower quadrant of the patient on PET/CT scans, which was strongly suggesting malignant tissue, with concomitant clinical signs and symptoms. The diagnostic use of FDG-PET has been established in colon cancer [3]. Tahara et al. [4] published data about the use of 18F-FDG PET in detecting abscesses in 2 patients in the late 1980s. Since then, numerous case reports have been published, but data from larger series are rare. Some inflammatory lesions, especially granulomatous ones, may be markedly FDG-avid. These include inflammatory bowel disease and abscesses [5]. In our case, we did not encounter an abscess within or around the peri-appendiceal mass. In addition, the method has been highly sensitive in imaging patients at a high risk of infection and of metastatic infectious disease, even when the results of other diagnostic procedures were normal [6,7]. However, this situation may cause false positive malignant tissue interpretations as in our case. However, Gutman [8] reported a 25% false positivity with FDG PET/CT uptake in 20 pts (21 foci), which have been proven by colonoscopy.

Annovazzi et al [9] evaluated the scintigraphic methods for the diagnosis of diverse intra-abdominal infective/inflammatory disorders. They have not been able to find any published matter about the application of PET/CT on the diagnosis of appendicitis in a peer review of 20 years evidenced base medicine screening (1984-2004).

FDG trapped tissues are analyzed semi-quantitatively using the standard uptake value (SUV), which relates the activity concentration in a fixed volume of tissue to the amount of the injected dose and the patient’s body weight. SUV characteristic of a tissue is as important as the uptake of the radiopharmaceutical. Gutman et al [8] found a difference in the mean SUV(max) between false positive and true-positive colonic FDG foci but not statistically significant (p = 0.14). They suggested that the presence of a focal colonic FDG uptake incidental finding on a PET/CT scan justifies a colonoscopy to detect (pre-) malignant lesions. In our case, early and late SUV values are 10.8 and 14.7 respectively, which are quite similar of a malignant tissue. A cut-off SUV value of 3 has been suggested in the past for the discrimination of benign and malign lesions but in the gastrointestinal tract variable FDG uptake means that benign lesions can have SUVs of between 5 and 10 [10]. Another study reported dual-point FDG-PET imaging for differentiating malignant from inflammatory processes [11]. The authors demonstrated that the SUV significantly increased in tumors overtime, whereas the SUV of inflammatory lesions decreased overtime. Imdahl [12] et al assumed a SUV of 4 as an indicator of possible malignant lesion which has taken on delayed image acquisition.

False positivity still seems a diagnostic dilemma of FDG-PET/CT. False positives may be reduced with the use of multitracer studies and labelled amino acids instead of the FDG scan [13]. A different approach using dual time point imaging was helpful in differentiating malignancy from inflammation and normal tissue in the head and neck, particularly when separated by a sufficient time interval of more than 30 minutes [14]. This method has found application in head and neck tumours but more studies are required to investigate its effectiveness in other cancers.

## Conclusion

Because FDG is a tracer of glucose metabolism, its distribution is not limited to malignant tissue. To avoid misinterpretations, the interpreter must be familiar with the normal pattern and physiologic variations of FDG distribution and with clinical data relevant to the patient. It still seems a diagnostic dilemma to differentiate a peri-appendicular mass due to an appendicitis from a malignant mass by means of PET/CT scan. Our case is a demonstration of a misinterpretation of colonic tumor due to its high metabolic rate. This clinical condition has emerged a need for further study on the diagnostic accuracy of PET/CT scan in the diagnosis of acute appendicitis and/or peri-appendicular mass.

## Abbreviations

CBC: Complete Blood Count
CT: Computerized Tomography
ER: Emergency Room
F18: Fluor 18 isotope
FDG: Fluorodeoxyglucose
FDG-PET: Fluorodeoxyglucose-Positrone Emission Tomography
PET: Positron Emission Tomography
SUV: Standard Uptake Value
USG: Ultrasonography
